# Production and characterization of poly(3-hydroxybutyrate-*co*-3-hydroxyvalerate) copolymers from a pre- fermented hardwood hydrolysate

**DOI:** 10.1007/s00449-025-03203-8

**Published:** 2025-07-18

**Authors:** Warren Blunt, Purnank Shah, Vinicio Vasquez, Mengwei Ye, Christopher Doyle, Yali Liu, Sajjad Saeidlou, Fanny Monteil-Rivera

**Affiliations:** 1 Aquatic and Crop Resource Development Research Centre, National Research Council Canada, 6100 Royalmount Avenue, Montreal, QC H4P 2R2 Canada; 2 FPInnovations, 570 Boulevard Saint-Jean, Pointe-Claire, QC H9R 3J9 Canada; 3 Automotive and Surface Transportation Research Centre, National Research Council Canada, 75 de Mortagne Boulevard, Boucherville, QC J4B 6Y4 Canada; 4 Present Address: Department of Biosystems Engineering, University of Manitoba, Winnipeg, MB R3T 5V6 Canada

**Keywords:** Lignocellulose, Polyhydroxyalkanoates, *Propionibacteria*, *Hydrogenophaga*, *Paraburkholderia*

## Abstract

Economically viable production of poly(3-hydoxybutyrate-*co*-3-hydroxyvalerate) (PHBV) copolymers remains a challenge. The objective of this work was to produce low-cost PHBV copolymers from lignocellulose-derived mixed sugars without genetic engineering or addition of chemical precursors. A hardwood hydrolysate was first pre-fermented using the facultative anaerobe *Propionibacterium acidipropionici,* and the resulting propionate-rich effluent was used for subsequent PHBV biosynthesis in *Paraburkholderia sacchari* or *Hydrogenophaga pseudoflava*. *P. acidipropionici* displayed a high tolerance to the hardwood hydrolysate and produced up to 11 g L^−1^ propionate (with varying amounts of lactate and acetate) under batch conditions. Propionic acid exerted significant toxicity toward *P. sacchari* and *H. pseudoflava*, so dilution of the pre-fermentation effluent was required prior to the PHBV production step. When *P. sacchari* and *H. pseudoflava* were grown on the pre-fermented mixture of glucose, xylose, lactate, acetate, and propionate (diluted to 15 mM propionate), the organic acids were consumed preferentially. *H. pseudoflava* accumulated up to 41.7 ± 7.0% cell dry mass (CDM) as PHBV that contained 13.7 ± 2.4 mol % 3-HV subunits. Meanwhile, *P. sacchari* accumulated up to 56.0 ± 5.8% CDM as PHA, but with lower 3-HV contents (1.2–5.1%). The PHBV copolymers resulting from this integrated process showed a desirable crystallinity, but the molecular weights were lower and the melt temperatures were higher than expected in all cases. Future work should focus on tuning the cultivation parameters to target higher molecular weight polymers while designing a feeding strategy of the pre-fermented stream that circumvents toxicity issues and allows a better control of the formation of random *vs*. block copolymers.

**Graphical abstract:**

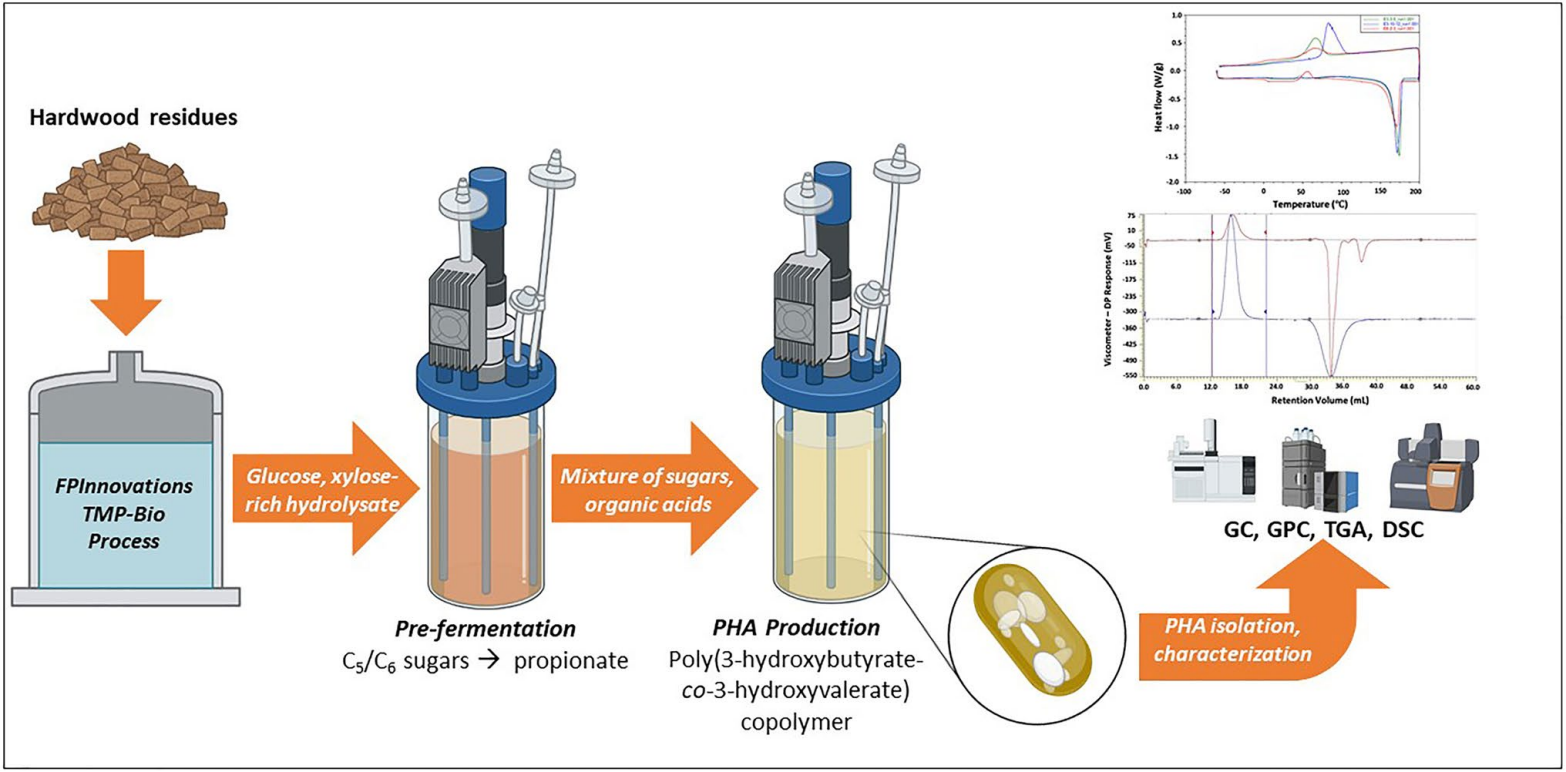

**Supplementary Information:**

The online version contains supplementary material available at 10.1007/s00449-025-03203-8.

## Introduction

There are growing calls to action for reducing plastic waste and mitigating its consequences on both the environment and human health [1]. The manufacture and application of biopolymers, such as microbial polyhydroxyalkanoates (PHAs), can make a positive contribution toward the fight against plastic pollution. PHAs are a class of bio-polyesters synthesized by certain bacteria and archaea as reserves of carbon and energy [2]. In addition to being biodegradable, they are also renewable since they can be produced from bio-sourced carbon [3] or autotrophically from CO_2_ [4].

Despite promise, there are several challenges associated with PHA commercialization, including the cost of production relative to petrochemical alternatives and limitations in the desired thermal and mechanical properties [5]. As much as 50% of the production cost is attributable to the cost of the carbon substrate [6]. This has resulted in significant attention toward utilizing low value “waste” carbon sources [7, 8] and coupling PHA production to the production of other value-added compounds in the context of a multi-product biorefinery [9, 10]. The many possible monomer subunit combinations [11], the composition and microstructure of which can affect the physical and thermal properties of the polymer, might also complicate the commercialization path [12, 13]. While the diversity of polymer structures, their properties, and consequent applications can be advantageous, controlling and tailoring the polymer properties to meet the specifications of a particular application remains challenging. Notably, poly(3-hydroxybutyrate) (PHB) is the most widely synthesized and well-studied PHA polymer but its highly crystalline nature makes it a hard and brittle polymer that is difficult to process [14]. There has been significant attention toward incorporation of co-monomers such as 3-hydroxyvalerate, 4-hydroxybutyrate, and 3-hydroxyhexanoate to improve the thermal and mechanical properties of the PHAs compared to a PHB homopolymer [15, 16].

With these challenges in mind, the goal of this work was to produce value-added copolymers from sugar mixtures derived from (renewable) lignocellulosic biomass. Canada has an abundance of forest resources that cover a land area of 309 Mha [17]. A possible source of lignocellulosic sugars is FPInnovations’ proprietary process (TMP-Bio) for the extraction of H-lignin and sugar co-streams from hardwood biomass [18, 19]. The sugar stream is a mixture of predominantly C_6_ (glucose) and C_5_ (xylose) sugars present at a ratio of approximately 2.5:1 [20]. Previously, it was shown in a comparison of three strains known to synthesize PHA from both glucose and xylose, that both *P. sacchari* and *H. pseudoflava* performed favorably when cultivated on TMP-Bio sugars compared to synthetic mixtures of commercial glucose and xylose in minimal medium [21]. In that work, *H. pseudoflava* showed particularly promising performance, accumulating up to 84% of the cell dry mass (CDM) as PHB resulting in a product titer of more than 5 g L^−1^ in batch (flask scale) cultivations. Moreover, the two strains could accumulate PHBV copolymers when a 3-HV precursor was added to the mixture of TMP-Bio sugars. *H. pseudoflava* showed a significantly higher propionate-to-3-HV yield than the other strains, and this also had a positive effect on the molecular weight of the polymer. The present study builds on that previous work by integrating a pre-fermentation step into the process as a source for propionic acid as a precursor to PHBV copolymer production. The pre-fermentation step was carried out with *P. acidipropionici*, a Gram-positive, non-spore-forming facultative anaerobe that was chosen for its ability to catabolize both C_5_ and C_6_ sugars to propionic acid. The subsequent mixtures of sugars and organic acids were then used to produce PHBV copolymers, and the resulting polymers were characterized for composition, molecular weight, and thermal properties.

## Materials and methods

### Strains

The production of volatile fatty acids (VFAs, specifically propionate) was accomplished using the facultative anaerobic bacterium *P. acidipropionici* ATCC 4875. This strain was revived in ATCC 2210 medium from glycerol stock cultures stored at − 80 °C. The PHA production step utilized *P. sacchari* DSM 17165 and *H. pseudoflava* ATCC 33688, which were revived and maintained as previously described [21].

### Carbon substrates

Unless otherwise specified, the carbon source was a hardwood hydrolysate produced from Aspen woodchips via the TMP-Bio process at FPInnovations (Canada) as detailed previously [18, 19]. In this proprietary process, the wood chips were first pretreated using a thermomechanical pulping process prior to chemical treatment, using a swelling agent, and enzymatic hydrolysis process using commercially available xylanase and cellulase enzymes [20]. The resulting hydrolysate had a total sugar concentration of ~ 130 g L^−1^ and consisted of mainly glucose and xylose at a ratio of approximately 2.5:1. The sugar solution was centrifuged, 0.22-µm filtered and stored at 4 °C prior to usage. The hydrolysate was diluted either tenfold or twofold for the pre-fermentation step.

### Media preparation

*P. acidipropionici* was revived on ATCC 2210 medium, which consists of (per L distilled water): 30 g trypticase, 20 g beef extract, 5 g yeast extract, 5 g KH_2_PO_4_, 1.0 mg vitamin K1, 5.0 mg hemin, 0.5 g L-cysteine HCl, and 4.0 mL of 0.025% resazurin. Two experiments were conducted to screen inexpensive alternatives to 2210 medium in the VFA production step, with the goal of making the pre-fermentation step more industrially attractive. These experiments included: i) testing growth and propionate synthesis in minimal media; and ii) testing the effect of substituting expensive complex components (trypticase, beef extract, yeast extract) of 2210 with corn steep solids (CSS). In the first set of experiments, a series of dilutions of 2210 medium into a mineral salt medium (MSM, described below) were prepared. These featured 2210 medium as the control, 1:4 and 1:10 (2210:MSM) dilutions, and MSM. For these tests, each medium was supplemented with TMP-Bio sugars as the carbon source at a total initial sugar concentration of ca. 11 g L^−1^. For the second test examining the use of CSS, MSM was used as the base medium with CSS (Corn Steep Liquor Powder, Marcor Development Corp) added in concentrations ranging from 0 to 30 g L^−1^. The total initial sugar concentration was set at ca. 70 g L^−1^ by mixing a twofold concentrated MSM with the liquid TMP-Bio sugar solution (~ 130 g L^−1^ total sugar concentration). These pre-fermentation experiments were conducted in 120 mL serum bottles with a 50 mL working volume. The 2210 medium (exclusive of sugars) was added, and the bottles were sealed with butyl rubber stoppers and aluminum crimp seals prior to being autoclaved at 121 °C for 20 min. After autoclaving and cooling, the appropriate volume of the TMP-Bio hydrolysate was added aseptically. Finally, the bottles were sparged with 0.22-µm filtered N_2_ for 10 min to remove oxygen and promote biosynthesis of propionate over microbial biomass since *P. acidipropionici* is a facultative anaerobe.

The medium used for the PHA production step was MSM, which was prepared as described previously [20] This medium consisted of (per L distilled water): 1.5 g KH_2_PO_4_, 4.45 g Na_2_HPO_4_·7H_2_O, 1 g (NH_4_)_2_SO_4_, 0.2 g MgSO_4_·7H_2_O, 0.01 g CaCl_2_·2H_2_O, 0.06 g ferric ammonium citrate, and 0.2 mL of a trace element solution. The composition of the trace element solution was (per L distilled water): 0.3 g H_3_BO_3_, 0.3 g CoCl_2_·6H_2_O, 0.2 g ZnSO_4_·7H_2_O, 0.2 g (NH_4_)_6_Mo_7_O_24_·4H_2_O, 0.02 g NiCl_2_·7H_2_O, 0.01 g CuCl_2_·2H_2_O, 0.03 g MnCl_2_·4H_2_O. The phosphate buffers and (NH_4_)_2_SO_4_ source were added and autoclaved, while filter-sterilized solutions of CaCl_2_, MgSO_4_, ferric ammonium citrate, trace elements, and the carbon source were added aseptically after the medium had cooled. Pre-fermented TMP-Bio liquor was used as the carbon source unless otherwise specified. All chemicals and products were purchased from Sigma Aldrich (Oakville, ON) unless specified otherwise.

### Cultivation conditions

#### VFA production

*P. acidipropionici* was revived from a glycerol stock in aerobic 2210 medium and incubated for 72 h. Experiments were initiated by inoculating the serum bottles to obtain a starting OD_600_ of 0.08 and monitored for 144 h. Samples (2 mL) were taken daily, aseptically, and handled as described below. The bottles were incubated at 30 °C without shaking. All serum bottle experiments with *P. acidipropionici* were performed in duplicate.

The final medium selected for VFA production in the serum bottle experiments was MSM supplemented with 20 g L^−1^ CSS. TMP-Bio sugars were added as the carbon source to have a total initial sugar concentration of ca. 70 g L^−1^. The pre-fermentation experiments were scaled up in a 2-L Eppendorf Dasgip bioreactor with a 1 L working volume to generate enough material to support downstream PHA production. The temperature was maintained at 30 °C and the pH was controlled at 6.8 via automatic addition of either 2N NaOH or 2N H_2_SO_4_. The reactor was sparged with N_2_ at a flow rate of 100 mL min^−1^ to maintain anaerobic conditions similarly to what was done in the bottle experiments. Constant mixing at 250 rpm was applied using a dual Rushton turbine. The bioreactor was inoculated to target a starting OD_600_ of 0.08 and monitored for 144 h; samples (2 mL) were withdrawn daily from the bioreactor and handled as described below.

Following the pre-fermentation step, the cells and residual CSS were removed by centrifugation (12,000 × *g* for 20 min) and supernatant was filter-sterilized using a 0.2 µm Millipore Stericup filter. The pre-fermented liquor was then stored at 4 °C until subsequent use in the PHA production experiments.

#### Minimum inhibitory concentration (MIC) tests

The MIC for each product from the VFA production step (propionate, lactate, acetate) was evaluated for both *H. pseudoflava* and *P. sacchari*, using baffled 250-mL flasks with a 50-mL working volume of MSM medium supplemented with 10 g L^−1^ commercial glucose as the main carbon source. Treatments were spiked with filter-sterilized 1 M aqueous solutions (pH adjusted to 7.0) of either propionate, acetate, or lactate. The concentrations for *P. sacchari* were varied from 0 to 80 mM, while those for *H. pseudoflava* were varied from 0 to 40 mM. *P. sacchari* and *H. pseudoflava* were incubated for 72 h at 125 rpm at 30 and 37 °C, respectively. The OD_600_ and pH were tracked throughout the cultivation and changes in the rate of increase in OD_600_ (representing combined growth and/or PHA synthesis) with respect to the control culture (10 g L^−1^ glucose in MSM) were used as indicators of inhibition. The final cultures (at 72 h) were harvested for residual sugars and PHA analysis as described below. A minimum of two biological replicates was performed for each condition in the MIC tests.

#### PHA production from pre-fermented TMP-Bio sugars

Considering the results from the MIC tests, the pre-fermented TMP-Bio liquor was diluted to have a propionate concentration of approximately 15 mM (1.11 g L^−1^). To support sufficient growth, the sugar concentration was adjusted to have an initial starting value of approximately 10 g L^−1^ glucose and 5 g L^−1^ xylose, similar to previous experiments [21]. Because of the relatively dilute sugar concentration in the TMP-Bio mixture, a slightly concentrated (1.25x) version of the medium was prepared to maintain the same buffering capacity and concentrations (*i.e*., C/N and C/P ratios) of the various medium components. The PHA production tests were performed in triplicate.

### Sample handling and biomass measurement

Following measurement of pH and OD_600_ during the various cultivations, each 2 mL sample was centrifuged at 16,000 × *g* for 5 min. The supernatant was stored at -20 °C for subsequent analysis and the pellet was discarded. At the end of the cultivations for PHA production, the remaining culture volume was centrifuged (12,000×*g* for 20 min). The cell pellet was washed in phosphate buffered saline (PBS), transferred to a pre-weighed aluminum tray, and oven dried at 70 °C for a minimum of 24 h.

### Analytical methods

#### Analysis of intracellular PHA content and monomer composition

Intracellular PHA content and monomer composition were determined as previously described [21] using gas chromatography coupled with flame ionization detection (GC-FID) after acid-catalyzed methanolysis [22].

#### PHA extraction, purification, and properties

PHA was extracted from the dried biomass using Soxhlet extraction with chloroform for 4 h, and purified by precipitation in cold methanol, as previously described [21]. The weight average (*Mw*) and number average (*Mn*) molecular weights, as well as the polydispersity index (PDI) of the purified polymers were determined by gel permeation chromatography (GPC) using a Viscotek GPCmax TDA 305 instrument coupled with triple detection (refractive index, viscosity, and light scattering) [21]. Thermal properties [degradation temperature (*T*_*d*_), melting temperature (*T*_*m*_), crystallization temperatures (*T*_*c*_), glass transition temperature (*T*_*g*_), melt enthalpy (Δ*H*_*m*_), and crystallization enthalpy (Δ*H*_*c*_)] of the polymers were also determined using either thermal gravimetric analysis (TGA) or differential scanning calorimetry (DSC), along with the instruments and protocols described previously [21].

#### Sugar and VFA analysis

Both sugars (glucose, xylose) and organic acids (acetate, lactate, propionate) were quantified by high-performance liquid chromatography (HPLC) using a refractive index detector. The HPLC unit consisted of a model 600 pump, model 717 Plus autosampler, and a model 2414 refractive index detector from Waters. Separation was performed using a Transgenomic ICSep ICE-ION-300 column (300 mm × 7.8 mm OD) maintained at 35 °C and a mobile phase of 0.01 N H_2_SO_4_ at a flow rate of 0.4 mL min^−1^. Thawed supernatant samples were centrifuged at 16,000 × g for 5 min and subsequently diluted in the mobile phase so that analytical concentrations did not exceed 10 g L^−1^.

### Statement of human and animal rights

This study did not involve any human or animal subjects.

## Results and discussion

### Medium screening for fermentation of TMP-Bio sugars to VFAs using* P. acidipropionici*

**Fig. 1:**
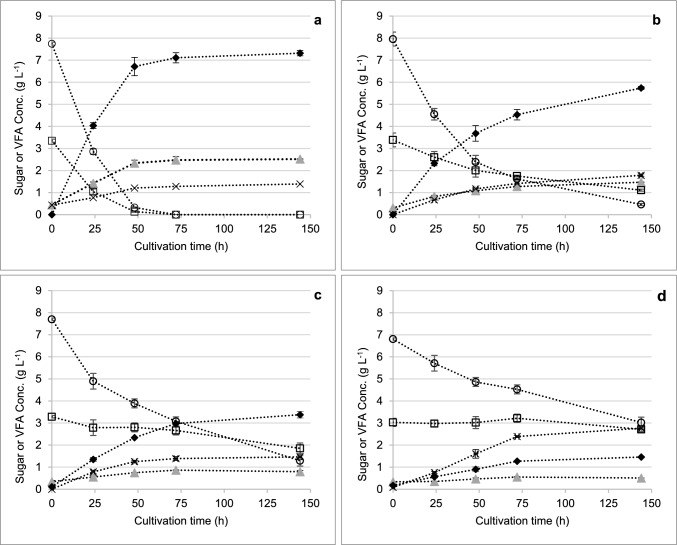
Mixed sugar uptake and VFA production from *P. acidipropionici* grown in different media supplemented with TMP-Bio sugars at an initial total sugar concentration of 11 g L^−1^. **a** 2210 medium; **b** 2210 medium diluted in MSM at 1:4; **c** 2210 medium diluted in MSM at 1:10; **d** MSM medium. Symbols: , acetate; ◆, propionate; ×, lactate; ○, glucose; ☐, xylose. Error bars represent standard deviations between biological duplicates

Synthesis of propionate using *P. acidipropionici* was first investigated in 2210 medium supplemented with TMP-Bio sugars. In this medium, the strain co-consumed approximately 11 g L^−1^ glucose and xylose to depletion within 72 h (Fig. 1a). At 144 h, a maximum of 7.32 g L^−1^ propionate was detected, resulting in a sugar-to-propionate conversion yield (*Y*_Pro/S_) of 0.65 g g^−1^ (Table 1). Acetate and lactate were also detected as end-products with final concentrations of 2.52 and 1.39 g L^−1^, respectively. The final OD_600_ and CDM reached values of 8.9 and 2.95 g L^−1^_,_ respectively. On the other hand, in MSM supplemented with TMP-Bio sugars, the maximum OD_600_ was reduced to 1.23 (0.47 g L^−1^ CDM) and only 3.8 g L^−1^ glucose and 0.3 g L^−1^ xylose were consumed (Fig. 1d). The final propionate titer was 1.46 g L^−1^ (*Y*_Pro/S_ = 0.36 g g^−1^), and the end-product profile also shifted such that lactate became the predominant end-product. In all cases, the majority of the fall in pH (from 7 to less than 5) occurred within the first 24 h of cultivation and was accompanied with a plateau in the OD_600_, although propionate synthesis continued generally for 72 h before reaching a plateau. The present findings confirm the observations made by Coral et al. [23], who reported the inhibition of *P. acidipropionici* growth at pH < 5; they also reinforced the fact that restricting biomass may be favorable for propionate production, as previously observed by others [24, 25]. The production of cell biomass, uptake of xylose and production of propionate were all reduced when *P. acidipropionici* was grown in minimal medium, which highlights the importance of having a source of complex nitrogen in the medium to effectively convert sugars into propionate. This is not unexpected given that all *Propionibacteria* are known to require both pantothenate (vitamin B5) and biotin (vitamin H) [26]. The comparatively low titer of propionate in MSM may be due to lower activity of the biotin-dependent methylmalonyl-CoA carboxytransferase, a key enzyme in synthesis of propionate via the Wood–Werkman cycle, in minimal medium.

**Table 1:** Sugar uptake and end-product synthesis at 144 h for *P. acidipropionici* grown in the indicated medium supplemented with TMP-Bio sugars at an initial total sugar concentration of 11 g L^−1^

Medium	Total sugar uptake (g L^−1^)	Propionate titer (g L^−1^)	Acetate titer (g L^−1^)	Lactate titer (g L^−1^)	*Y*_Pro/S_ (g g^−1^)
2210	11.28 ± 0.00	7.31 ± 0.12	2.52 ± 0.01	1.39 ± 0.05	0.65 ± 0.01
2210:MSM (1:4)	9.77 ± 0.73	5.74 ± 0.07	1.47 ± 0.01	1.78 ± 0.07	0.59 ± 0.05
2210:MSM (1:10)	7.84 ± 0.69	3.37 ± 0.15	0.79 ± 0.06	1.47 ± 0.13	0.43 ± 0.06
MSM	4.10 ± 0.50	1.46 ± 0.01	0.51 ± 0.00	2.77 ± 0.06	0.36 ± 0.05

Tolerances represent standard deviations between a minimum of two biological replicates

*Y*_Pro/S_: yield coefficient of propionate per sugar consumed during the pre-fermentation step

**Fig. 2:**
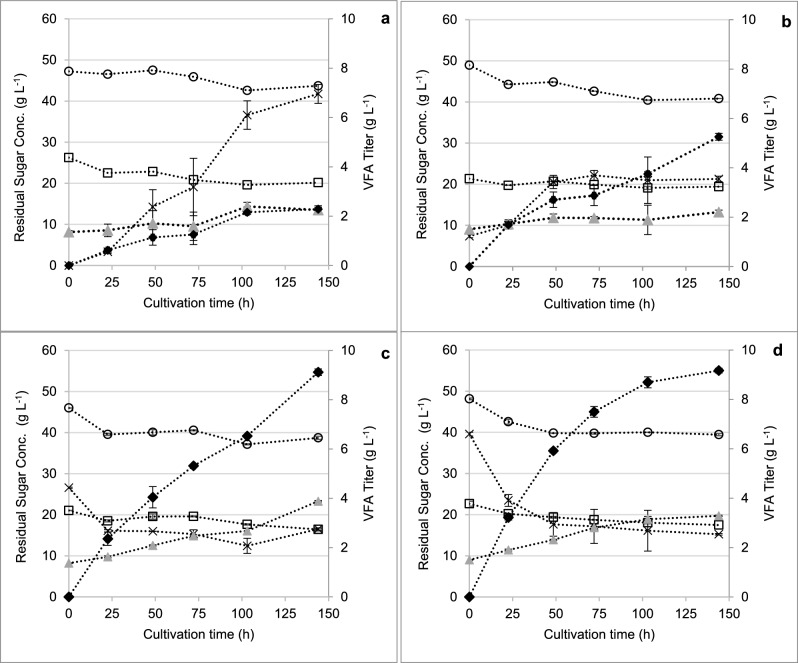
Mixed sugar uptake and VFA production from *P. acidipropionici* grown in MSM medium supplemented with TMP-Bio sugars at a total sugar concentration of ca. 70 g L^−1^. **a** 0 g L^−1^ CSS; **b** 5 g L^−1^ CSS; **c** 20 g L^−1^ CSS; and **d** 30 g L^−1^ CSS. Symbols: , acetate; ◆, propionate; ×, lactate; ○, glucose; ☐, xylose. Error bars represent standard deviations between biological duplicates

CSS has been used as an inexpensive source of nitrogen, carbon, and vitamins in various biotechnology processes [27]. Given the importance of a complex nitrogen source in the medium of present experiments, the use of CSS at different concentrations in MSM was investigated as an inexpensive nutritional alternative to 2210 medium. The propionate titer increased to a maximum of 11 g L^−1^ in MSM supplemented with 20 g L^−1^ CSS (Fig. 2 and Table 2). In this condition, the strain consumed 8.1 g L^−1^ glucose and 4.0 g L^−1^ xylose, resulting in a *Y*_Pro/S_ of 0.79 g g^−1^. This yield was slightly higher than that observed in 2210 medium, therefore supporting the applicability of CSS for propionate production using *P. acidipropionici*. The concentration of acetate remained relatively constant at 2–4 g L^−1^ among the different conditions (Fig. 2), whereas the initial lactate concentration increased with the initial CSS concentrations. This was indicative of acetate and lactate being present in the TMP-Bio sugars and CSS, respectively, which was confirmed by HPLC characterization of each substrate. At higher CSS concentrations, the concentration of lactate initially detected in the medium was as high as 6.6 g L^−1^, but subsequently decreased over time as shown in Fig. 2. Since lactate is catabolized to propionate in *P. acidipropionici *via the Wood–Werkman (or succinate) pathway, glycolysis and the ATP gain by substrate-level phosphorylation are by-passed compared to sugar metabolism [28, 29]. Thus, the presence of lactate as a medium component may promote propionate synthesis over biosynthetic reactions. This is supported by previous work in which Coral et al. [23] reportedly obtained the highest productivity using lactate among several carbon sources tested. In *Propionibacterium freudenreichii*, it has been reported that the presence of lactate induces changes to pyruvate metabolism and increases flux to propionate [30]. Overall, the current experiments using MSM supplemented with CSS indicated that: i) CSS could be used as a 2210 medium substitute; and ii) adding 20 g L^−1^ CSS to MSM was sufficient to give higher propionate titer and *Y*_*Pro/S*_ compared to 2210 medium. This is consistent with the range of concentrations for complex nitrogen sources reported in other studies [29].

**Table 2:** Sugar uptake and product synthesis for *P. acidipropionici* grown in MSM supplemented with TMP-Bio sugars at an initial total sugar concentration of ca. 70 g L^−1^ and the indicated CSS concentration

Concentration of CSS (g L^−1^)	Total sugar uptake (g L^−1^)	Propionate titer (g L^−1^)	Acetate titer (g L^−1^)	Lactate titer (g L^−1^)	*Y*_Pro/S_ (g g^−1^)
0	13.93 ± 2.62	1.67 ± 0.01	1.74 ± 0.03	4.30 ± 0.03	0.12 ± 0.02
5	11.12 ± 3.73	4.61 ± 0.24	2.04 ± 0.08	2.61 ± 0.17	0.44 ± 0.12
20	11.87 ± 3.02	11.05 ± 0.02	3.88 ± 0.03	2.75 ± 0.04	0.79 ± 0.01
30	13.86 ± 0.18	9.17 ± 0.10	3.28 ± 0.08	2.54 ± 0.03	0.66 ± 0.00

*Y*_Pro/S_: yield coefficient of propionate per sugar consumed during the pre-fermentation step

### VFA production in a bioreactor environment

MSM supplemented with 20 g L^−1^ CSS was chosen for VFA production in a bioreactor environment. The key hypothesis in this step was whether the addition of pH control could alleviate pH-induced inhibition and improve the propionate titer, while also producing enough of the pre-fermented hardwood hydrolysate to be used for subsequent PHA production. Over a 144-h bioreactor cultivation, the strain consumed over 40 g L^−1^ glucose and 10 g L^−1^ xylose (Supplementary Information Fig. S1), which corresponded to about fourfold and twofold higher sugar consumption compared to the serum bottle experiments with a similar medium (Fig. 2c, Table 2). Despite higher sugar consumption, increase in propionate titer was not observed, reaching a final concentration of 9.59 g L^−1^. The VFA profile in the bioreactor cultivation shifted from serum bottle experiments. As compared to the same medium in serum bottle experiments (Fig. 2c), lactate was produced over the cultivation and increased from an initial value of 3.5 g L^−1^ to 14.9 g L^−1^. This made lactate the predominant product in the bioreactor experiment as the resulting *net* production (11.4 g L^−1^) was higher than propionate. The acetate concentration, initially at 1 g L^−1^, increased to a final value of 2.9 g L^−1^ at 144 h. Finally, the higher consumption of sugars observed over the 144-h cultivation resulted in a *Y*_*Pro/S*_ of 0.19 g g^−1^, which was significantly lower than the values observed in the serum bottle experiments and shown in Tables 1 and 2. The propionate yield coefficients obtained in this work were generally consistent with previously reported values of 0.70–0.71 g g^−1^ in studies focusing on *P. acidipropionici* [31] and *P. freudenreichii* [32]. The obtained propionate titers and corresponding productivity values were, however, significantly lower than previous literature values using engineered strains of *P. acidipropionici* and cell recycling techniques, where optimized processes produced up to 106 g L^−1^ propionate [31] and volumetric productivities of nearly 3 g L^−1^ h^−1^ [33], respectively.

### Tolerance of *P. sacchari *and *H. pseudoflava* to VFAs synthesized by *P. acidipropionici*

With lactate and acetate being established as major end-products in addition to propionate in *P. acidipropionici* fermentations, the tolerance of the PHA-producing strains (*P. sacchari* and *H. pseudoflava)* to each of these fermentation end-products was explored via a series of tests to establish the MIC thresholds. There was no observable inhibitory effect of lactate on the OD_600_ profile for either *P. sacchari* or *H. pseudoflava* compared to the control condition of MSM supplemented with glucose as the carbon source (Supplementary Information Figs. S2 and S3). Lactate was depleted below detectable limits in nearly all cases except for *P. sacchari* at 80 mM lactate (7.21 g L^−1^), where traces (< 0.1 mM) were detected at 72 h. PHA synthesis was not inhibited by the presence of lactate at any concentration for either strain. Acetate was more toxic to both strains but the rate of biomass synthesis (represented as the rate of increase in OD_600_) remained above half the value observed in the control (Supplementary Information Figs. S4 and S5). For *H. pseudoflava*, the acetate was consumed below detectable limits in all conditions (0–40 mM), whereas < 1 mM residual acetate was detected in *P. sacchari* cultures for initial concentrations above 60 mM (3.60 g L^−1^). Biomass and intracellular PHA content were not adversely affected for acetate concentrations up to 60 mM in *P. sacchari* but declined significantly at 80 mM acetate (4.80 g L^−1^). For *H. pseudoflava*, the presence of acetate incrementally lowered both biomass and intracellular PHA content over the range of tested concentrations.

**Fig. 3:**
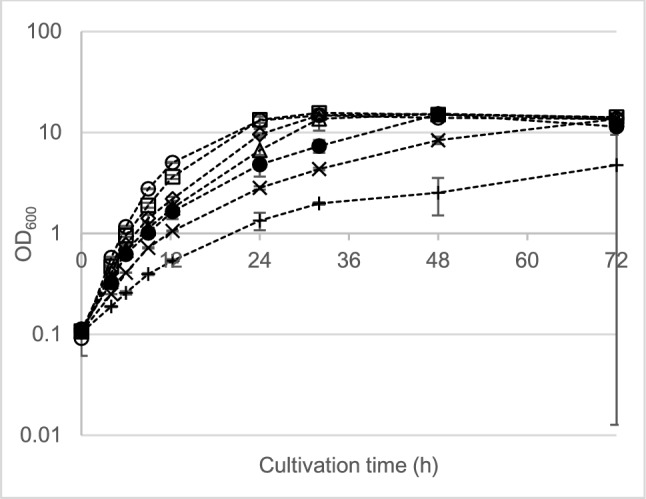
Biomass production (represented by OD_600_) in MSM with 10 g L^−1^ glucose supplemented with different propionic acid concentrations for *P. sacchari.* Symbols: ○, 0 mM (control); ☐, 10 mM; ◇, 20 mM; △, 30 mM; ●, 40 mM;  ×, 60 mM; +, 80 mM. Error bars represent standard deviations between a minimum of two biological replicates

**Fig. 4:**
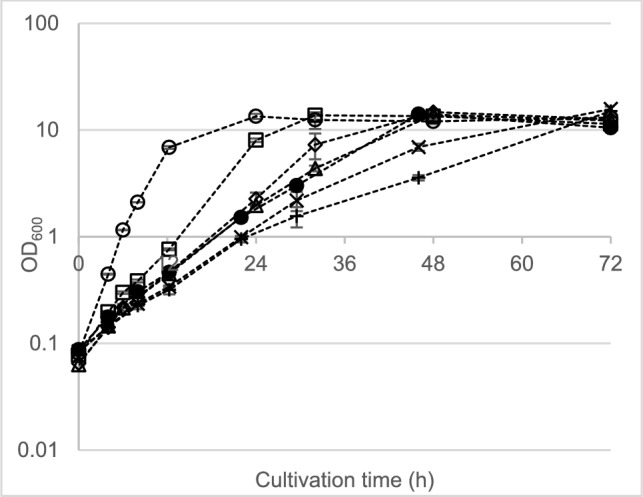
Biomass production (represented by OD_600_) in MSM with 10 g L^−1^ glucose supplemented with different propionic acid concentrations for *H.* pseudoflava. Symbols: ○, 0 mM (control); ☐, 5 mM; ◇, 7.5 mM; △, 10 mM; ●, 15 mM;  ×, 20 mM; +, 30 mM. Error bars represent standard deviations between a minimum of two biological replicates

The MIC tests with propionate showed higher toxicity to the strains than either lactate or acetate. Regardless, propionate was depleted below detectable limits in nearly all tests except for *P. sacchari* at 80 mM (5.93 g L^−1^), where the replicates showed inconsistent growth and visible signs of stress. This is indicated by the large standard deviations in this condition (Fig. 3). The rate of total biomass synthesis for *P. sacchari* decreased with increasing propionate concentrations (Supplementary Information Fig. S6). The intracellular PHA content and titer improved with increasing propionate concentration up to 20 mM (1.48 g L^−1^), where maximum values of 40.6% CDM and 1.86 g L^−1^, respectively, were obtained. At higher concentrations, both parameters showed a general decline (Supplementary Information Fig. S7). Propionate was significantly more inhibitory to *H. pseudoflava* than it was to *P. sacchari*. At concentrations of only 5 mM (0.37 g L^−1^), the rate of increase in OD_600_ was less than half its maximum value observed in the glucose-only control (Fig. 4). The maximum values for final total biomass (4.38 g L^−1^) and PHA titer (1.49 g L^−1^) were observed at 20 mM, but the highest intracellular PHA content was measured at 5 mM (44.7% CDM). By comparison, Bertrand et al. [34] reported that inhibition of growth and PHA synthesis in *H. pseudoflava* (formerly *Pseudomonas pseudoflava*) began at a concentration of 1 g L^−1^ (13.5 mM) propionate, with complete inhibition observed at 3 g L^−1^ (40 mM). In that work, the reported optimum concentration of propionate for PHBV titer in shake flask experiments was 0.3 g L^−1^, but the polymer contained only 3.5 mol% 3-HV subunits [34].

Analysis of the monomer composition of the PHA polymers obtained in the MIC tests revealed some key differences in copolymer production in each strain. In *P. sacchari* cultures supplemented with propionate, the 3-HV content increased up to 8.4 mol % at 40 mM propionate, after which little improvement was observed (Supplementary Information Fig. S7). A maximum propionate-to-3-HV yield coefficient (*Y*_*3-HV/Pro*_) of 0.02 g g^−1^ was obtained at a propionate concentration of 20 mM. Although *P. sacchari* showed a comparatively high tolerance to propionate, *H. pseudoflava* showed higher 3-HV content in the obtained polymers along with higher *Y*_*3-HV/Pro*_. The 3-HV content of *H. pseudoflava* polymers increased with initial propionate concentration, up to a maximum of 40.4 mol % 3-HV observed at 30 mM propionate (Supplementary Information Fig. S8). Compared to *P. sacchari*, this resulted in a relatively high *Y*_*3-HV/Pro*_ of 0.11 g g^−1^, which was observed at an initial concentration of 7.5 mM propionate. Although this value is still well below the theoretical maximum *Y*_*3-HV/Pro*_ of 1.35 g g^−1^ [35], this observation suggests the medium recipe could be tailored toward PHBV copolymers with predictable 3-HV composition using *H. pseudoflava*.

Short-chain VFAs like propionate exert toxicity to bacteria through disrupting cell membranes, uncoupling proton motive force, and altering intracellular pH [36, 37], although there is also evidence suggesting that propionate (or its catabolic intermediates) may alter gene expression [38]. According to Rocco and Escalante-Semerena [38], the best strategy to cope with high propionate concentrations is to efficiently catabolize it, which primarily involves the 2-methylcitrate pathway in many aerobic organisms. Pereira et al. [39] demonstrated that in *P. sacchari* mutants featuring a disrupted 2-methylcitrate pathway, the yield of 3-HV polymer subunits from propionate increased from 0.09 g g^−1^ in the wild type to 0.81–0.96 g g^−1^ in the mutant strains. In this work, the lower tolerance and higher 3-HV content and *Y*_*3-HV/Pro*_ observed in *H. pseudoflava* suggest that this organism has lesser ability to catabolize propionate than does *P. sacchari*. Weakening propionate catabolism could, therefore, be a strategy to obtain copolymers with higher 3-HV content [39, 40]; such a strategy would have to be carefully implemented to preserve the organism’s ability to detoxify its environment.

### Biosynthesis of PHBV copolymers from pre-fermented TMP-Bio

**Fig. 5:**
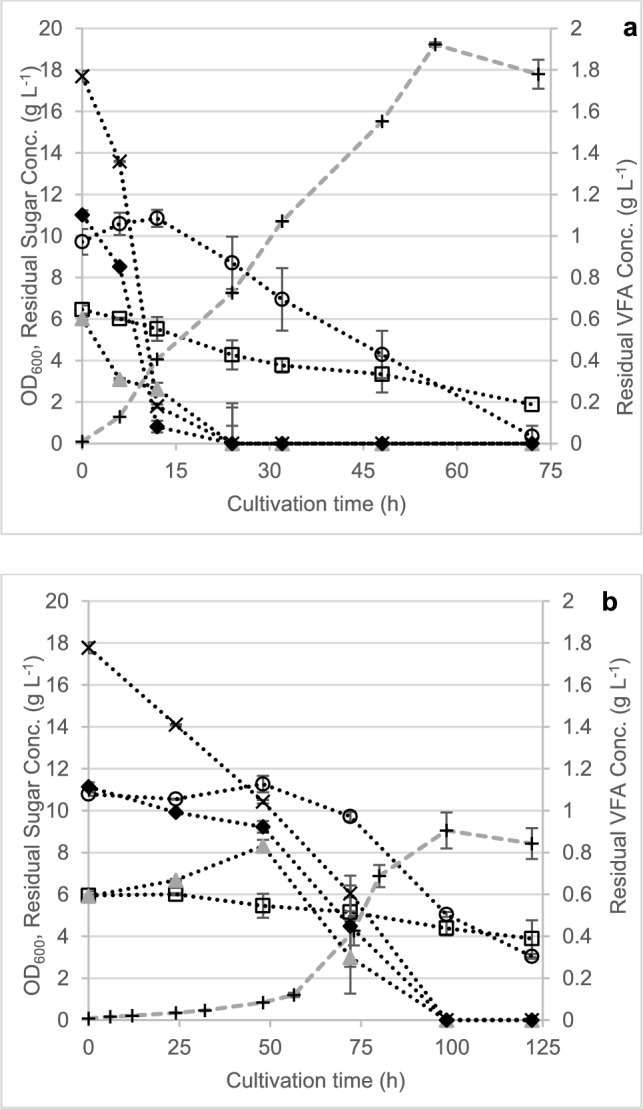
Growth and substrate uptake profiles for **a** *P. sacchari* and **b** *H. pseudoflava* in diluted effluent produced from pre-fermentation of TMP-Bio sugars with *P. acidipropionici*. Symbols: ▲, acetate; ◆, propionate;  ×, lactate; ○, glucose; ☐, xylose; +, OD_600_. Error bars represent standard deviations between biological triplicates

Knowing the concentrations of the different VFAs tolerable to each strain, both *P. sacchari* and *H. pseudoflava* were grown on the VFA-sugar mixture produced from fermentation of the TMP-Bio sugars with *P. acidipropionici* in a bioreactor. Based on the response of *H. pseudoflava* to propionic acid concentration in terms of both growth and PHA synthesis, an initial propionate concentration of 15 mM (1.1 g L^−1^) was targeted when diluting the pre-fermented TMP-Bio mixture. The same dilution was used for *P. sacchari* to ensure a similar C/N ratio for comparison. Biomass synthesis and substrate uptake profiles are shown in Fig. 5 for both *P. sacchari* and *H. pseudoflava*. *P. sacchari* reached a maximum OD_600_ of 19.2 at 57 h. Propionate, acetate, and lactate were all consumed within the first 24 h, and this was accompanied by a pH increase from 6.5 to 7.5. *H. pseudoflava* showed slower growth and carbon uptake compared to *P. sacchari* under these conditions so the cultivation was extended to 122 h. *H. pseudoflava* reached a maximum OD_600_ of 9.1 at 98.5 h, which also corresponded to the time when all three VFAs were depleted below detectable limits. Residual glucose and xylose were available throughout the cultivation in both cases, suggesting that both strains prefer the three organic acids as carbon source over either glucose or xylose.

PHA synthesis characteristics are shown in Table 3. *P. sacchari* accumulated higher intracellular PHA (50.7% CDM observed at 72 h) content and titer (2.7 g L^−1^) in a shorter time, resulting in higher overall productivity. Meanwhile, *H. pseudoflava* achieved lower content (41.7% CDM) and titer (1.6 g L^−1^) over the 122-h cultivation. As in the MIC tests,  *H. pseudoflava* was more effective at storing propionate as 3-HV subunits within the PHA polymer. From an initial concentration of 15 mM propionate, the final polymer at 122 h contained 13.7 mol % 3-HV subunits (Table 3). The polymer synthesized by *P. sacchari* contained only 1.2 mol % 3-HV subunits at 72 h. Since the HPLC analysis showed that propionate was consumed within the first 24 h, it was hypothesized that the polymer synthesized earlier in the cultivation would have higher 3-HV content. Another experiment with an earlier harvest time (22 h) confirmed the higher proportion of 3-HV at shorter cultivation times (5.1 mol %, Table 3) but this was obtained at the expenses of both intracellular PHA content and titer. Regardless of the harvest time, the maximum *Y*_*3-HV/Pro*_ for *P. sacchari* was only 0.03 g g^−1^, which is still threefold lower than the maximum *Y*_*3-HV/Pro*_ value (0.1 g g^−1^) observed in tests using *H. pseudoflava.*

**Table 3:** PHA synthesis and molecular weight characteristics of *P. sacchari* and *H. pseudoflava* from TMP-Bio sugars pre-fermented with *P. acidipropionici*

Strain	CDM (g L^−1^)	PHA Content (% CDM)	PHA titer (g L^−1^)	3-HV content (mol %)	*Y*_*3-HV/Pro*_ (g g^−1^)	*Mn* (Da)	*Mw* (Da)	*PDI*
*P. sacchari* (72 h)	5.42 ± 0.12	50.7 ± 0.4	2.7 ± 0.1	1.2 ± 0.1	0.02 ± 0.01	236,343	400,523	1.70
*P. sacchari* (22 h)	2.71 ± 0.06	26.0 ± 5.8	0.7 ± 0.1	5.1 ± 0.0	0.03 ± 0.01	231,203	372,946	1.61
*H. pseudoflava* (122 h)	3.37 ± 0.34	41.7 ± 7.0	1.4 ± 0.4	13.7 ± 2.4	0.10 ± 0.00	150,291	267,642	1.78

*Y*_3-HV/Pro_: yield coefficient of 3-HV co-monomer units per propionate consumed during the PHA production step, *M*_*n*_: number average molecular weight, *M*_*w*_: weight average molecular weight, *PDI*: polydispersity index

Copolymer production via addition of precursors was studied previously in *P. sacchari* to produce PHBV using propionic acid [41–43], valeric acid [43], levulinic acid [44], and γ-butyrolactone [45]. Meanwhile, in *H. pseudoflava*, Choi et al. [46] previously produced PHBV with 61 mol % 3-HV using 3% (vol vol^−1^) valerolactone. Although the maximum 3-HV contents reported in the literature for these strains are higher than those observed in the present study, in this work, the cultivation process and feeding strategy were not optimized and additional propionate feeding could have further improved the 3-HV content. As discussed above, weakening flux through propionate-catabolizing pathways could yield a polymer with higher 3-HV content. However, given the substrate preference observed in this work for VFAs, designing a cultivation strategy in which propionate is fed over time could allow for polymers with higher 3-HV contents and improved tunability toward either block or a randomly distributed copolymer, while also by-passing the toxicity issue.

### Physicochemical properties of PHBV copolymers synthesized from pre-fermented TMP-Bio sugars

The physicochemical properties of polymers, particularly their thermal and mechanical properties and molecular weight distribution, have large implications on the type of application they can be used for. PHB homopolymers are known to show a high degree of stiffness and brittleness as well as high melting temperature (*T*_*m*_), close to their decomposition temperature (*T*_*d*_), that makes them hard to be processed using melt processes [47]. The incorporation of 3-HV into PHB usually leads to polymers that are less brittle, more ductile, tougher, and therefore better suited for industrial applications [47–49]. Incorporation of a higher fraction of randomly distributed 3-HV co-monomer along the polymer chains is also expected to disrupt their packing and crystalline nature and lead to reductions in *T*_*m*_. The resulting increased separation of the *T*_*m*_ from the *T*_*d*_ is an expected benefit of the copolymers from a processing standpoint [13, 50].

Although intuitively one would expect to see linear relationships between the amounts of 3-HV units incorporated in the copolymers and their thermal properties, several previous reports indicated that there are other factors influencing thermal properties of polymers besides monomer compositions and monomer amounts [47, 48]. Physicochemical properties have, thus, been measured herein for the various copolymers produced in *P. sacchari* or *H. pseudoflava*.

The polymer molecular weight is a key characteristic determining its utility and application. For polymers to be processable at a commercial scale, it is recommended that the molecular weight be at least 400–500 kDa (higher values for thermoplastic applications) while maintaining a PDI of less than 3 [13, 51, 52]. The *Mw* of the PHBV copolymers produced using *P. sacchari* or *H. pseudoflava* grown on pre-fermented TMP-Bio sugars were 400.5 and 267.6 kDa, respectively (Table 3). The PDIs (1.70–1.78) obtained with both strains were indicative of relatively uniform polymer chains. When these strains were grown on TMP-Bio sugars in minimal medium spiked with commercial propionate, lower PDI values (1.19–1.45) and higher *Mw* values were obtained for *P. sacchari* (516.7 kDa) and *H. pseudoflava* (539–1,268 kDa) [21]. In that previous work, the same strains and otherwise similar medium and cultivation conditions were used, so the observed differences in the molecular weight and polydispersity in the current study were attributed to the pre-fermentation step. In this regard, the major differences are medium carry over from the pre-fermentation step used in this study, which contained soluble fractions left from the CSS used to promote growth of *P. acidipropionici,* and the presence of organic acids (acetate and lactate) co-produced with propionate. Further studies would be necessary to identify the relationship between the nature of medium, the co-presence of organic acids such as lactate, and the molecular weights of produced PHAs in these strains. Once the relationship is clarified, one might need to tune the pre-fermentation step to prevent the presence of co-factors affecting negatively the molecular weight of the final PHBV. Environmental parameters like temperature [53], pH [54], and oxygenation levels [55] should also be tested as they have all been shown to impact the polymer molecular weight, through alteration of catalytic activity of the PHA synthase or depolymerase [56]. Another approach to increase molecular weights could be strain engineering strategies such as knocking out the PHA depolymerase enzyme [57, 58].

The thermal characteristics of the polymers produced by *P. sacchari* and *H. pseudoflava* are shown in Table 4. The polymers produced by either strain had similar *T*_*d*_ values, in the range of 290–293 °C. With progressively higher 3-HV content, reductions in *X*_*c*_, *T*_*g*_, and Δ*H*_*m*_ were observed in each PHBV sample, representing an increasingly amorphous nature (Table 4). For comparison, previous work in which PHB homopolymers were produced from TMP-Bio sugars in these same strains showed *T*_*g*_, and *X*_*c*_ values in the range of 1.6–5.4 °C, and 57%, respectively [21]. Despite producing copolymers that contained up to 13.7 mol% 3-HV subunits, a significant reduction in peak *T*_*m*_ was not observed compared to commercial PHB. The obtained *T*_*m*_ values were only slightly lower than for PHB produced from TMP-Bio sugars in these strains, reported previously as ranging within 171–172 °C [21]. Similar observations have been reported where *T*_*m*_ of PHBV remained unchanged despite different contents of 3-HV [47, 48].

**Table 4:** Thermal properties of PHBV copolymers synthesized by *P. sacchari* and *H. pseudoflava* from TMP-Bio sugars pre-fermented with *P. acidipropionici*

Strain	3-HV (mol%)	*T*_*d*_ (°C)	*T*_*m*_ (°C)	Δ*H*_*m*_ (J/g)	*T*_*g*_ (°C)	*T*_*c*_ (°C)	Δ*H*_*c*_ (J/g)	*X*_*c*_ (%)
*P. sacchari* (72 h)	1.2	292.4	169.7	83.1	2.7	90.0	63.7	56.9
*P. sacchari* (22 h)	5.1	290.9	170.4	64.8	− 0.8	56.1	19.4	44.4
*H. pseudoflava* (122 h)	13.7	293.1	170.3	59.9	− 11.6	76.6	39.8	41.0
Commercial PHB	0	294.9	161.8	82.8	2.8	81.7	61.0	56.7
Commercial PHBV	8	290.0	143.5	61.3	− 6.8	103.0	55.0	42.0

*T*_*d*_: thermal degradation temperature, *T*_*m*_: melting temperature, Δ*H*_*m*_: melt enthalpy, *T*_*g*_: glass transition temperature, *T*_*c*_: crystallization temperature, Δ*H*_*c*_: crystallization enthalpy, *X*_*c*_: crystallinity index

Comparing the thermal properties of the two polymers synthesized by *P. sacchari vs.* the one obtained with *H. pseudoflava* suggests that the two types of polymers have differences in microstructures (Supplementary Information Figs. S9-S11). In the case of *P. sacchari*, an increase of the 3-HV from 1.2 to 5.1% resulted in a significant reduction in *T*_*c*_ from 90 to 56 °C, a reduction in Δ*H*_*c*_ from 63.7 to 19.4 J/g, and the appearance of a cold-crystallization peak upon subsequent heating. This confirms that the 3-HV monomers impede the crystal nucleation and growth, supporting a uniform distribution of 3-HV within the polymer chains. A possible explanation for the two *Tm* peaks appearing in the melt endotherm is that the formation of large crystals was still possible, even to a low extent, at the concentrations of the 3-HV obtained (Fig. S10). On the other hand, the microstructure of the chains of the PHA synthesized by *H. pseudoflava* was closer to a block copolymer (Fig. S11), resulting in domains richer in 3-HV with lower melting point and domains with less co-monomers that could crystallize at a higher rate and melt at a higher temperature. Although both copolymers had their highest *T*_*m*_ close to 170 °C, the fact that their thermal decomposition temperatures were around 290 °C suggests a processing window wide enough to allow workability of both polymers using melting processes.

## Conclusion

The aim of this work was to address the challenges of cost reduction in PHA biopolymer production as well as improve the polymer properties through process design and integration. *P. acidipropionici* was used to pre-ferment a C_5_ and C_6_ sugar stream derived from Canada’s forestry sector into precursor molecules (propionic acid) for PHBV copolymer synthesis. In the presence of corn steep solids as an inexpensive medium for *P. acidipropionici*, the strain produced up to 11 g L^−1^ propionate in batch conditions. The resulting mixture of organic acids and residual sugars was then converted to PHBV copolymers using *P. sacchari* or *H. pseudoflava*, which have previously demonstrated potential to convert xylose, glucose, and propionate into PHBV. *P. sacchari* demonstrated better tolerance and growth to the propionate in the pre-fermented effluent and produced a higher PHA titer than *H. pseudoflava*, but the latter strain was considerably more effective at converting the available propionate into 3-HV monomers within a PHBV copolymer. *H. pseudoflava* produced a copolymer with up to 13.7% 3-HV monomer subunits and while this showed a reduction in *T*_*g*_, Δ*H*_*m*_, and *X*_*c*_, the *T*_*m*_ was not significantly reduced compared to a PHB homopolymer and the molecular weight was lower compared to previous work with these strains without the pre-fermentation step. While the process showed promise, the results indicate sensitivity of the polymer properties to the strain and composition of the medium used as well as limitations due to inhibition when using a batch-wise addition of copolymer precursors. We recommend targeting two central aspects for future work, including: (i) elucidating the effects of medium composition and process control parameters (pH, dissolved oxygen) on polymer molecular weight to favor the production of PHBV of higher molecular weights; as well as (ii) designing a feeding strategy for the pre-fermented effluent to circumvent toxicity issues obtained using batch-wise addition, while tuning the 3-HV content and distribution in the copolymer.

## Supplementary Information

Below is the link to the electronic supplementary material.

Supplementary file1 (DOCX 247 KB)

## Data Availability

No datasets were generated or analyzed during the current study.

## References

[1] Han M, Liu H, Zhu T et al (2024) Toxic effects of micro(nano)-plastics on terrestrial ecosystems and human health. TrAC Trends Anal Chem 172:117517. 10.1016/j.trac.2023.117517

[2] Mukherjee A, Koller M (2023) Microbial polyhydroxyalkanoate (PHA) biopolymers—intrinsically natural. Bioengineering 10:855. 10.3390/bioengineering10070855 37508882 10.3390/bioengineering10070855 PMC10376151

[3] Mahato RP, Kumar S, Singh P (2023) Production of polyhydroxyalkanoates from renewable resources: a review on prospects, challenges and applications. Arch Microbiol 205:172. 10.1007/s00203-023-03499-8 37017747 10.1007/s00203-023-03499-8

[4] De Wever H, Garcia-Gonzalez L (2023) Microbial Processes: Production of Polyhydroxyalkanoates from CO2. In: Kircher M, Schwarz T (eds) CO_2_ and CO as feedstock. Springer, Cham, pp 159–164

[5] Liang X, Cha DK, Xie Q (2024) Properties, production, and modification of polyhydroxyalkanoates. Resour Conserv Recycl Adv 21:200206. 10.1016/j.rcradv.2024.200206

[6] Koller M, Maršálek L, de Sousa Dias MM, Braunegg G (2017) Producing microbial polyhydroxyalkanoate (PHA) biopolyesters in a sustainable manner. New Biotechnol 37:24–38. 10.1016/j.nbt.2016.05.001 10.1016/j.nbt.2016.05.001 27184617

[7] Katagi VN, Bhat SG, Paduvari R et al (2023) Waste to value-added products: an innovative approach for sustainable production of microbial biopolymer (PHA)—emphasis on inexpensive carbon feedstock. Environ Technol Rev 12:570–587. 10.1080/21622515.2023.2250066

[8] Yukesh Kannah R, Dinesh Kumar M, Kavitha S et al (2022) Production and recovery of polyhydroxyalkanoates (PHA) from waste streams—a review. Biores Technol 366:128203. 10.1016/j.biortech.2022.128203 10.1016/j.biortech.2022.128203 36330969

[9] Yadav B, Talan A, Tyagi RD, Drogui P (2021) Concomitant production of value-added products with polyhydroxyalkanoate (PHA) synthesis: a review. Bioresour Technol 337:125419. 10.1016/j.biortech.2021.125419 34147774 10.1016/j.biortech.2021.125419

[10] González-Rojo S, Díez-Antolínez R (2023) Production of polyhydroxyalkanoates as a feasible alternative for an integrated multiproduct lignocellulosic biorefinery. Bioresour Technol 386:129493. 10.1016/j.biortech.2023.129493 37460022 10.1016/j.biortech.2023.129493

[11] Koller M, Mukherjee A (2022) A new wave of industrialization of PHA biopolyesters. Bioengineering 9:74. 10.3390/bioengineering9020074 35200427 10.3390/bioengineering9020074 PMC8869736

[12] Laycock B, Arcos-Hernandez MV, Langford A et al (2014) Thermal properties and crystallization behavior of fractionated blocky and random polyhydroxyalkanoate copolymers from mixed microbial cultures. J of Appl Polym Sci 131:app.40836. 10.1002/app.40836

[13] Mai J, Kockler K, Parisi E et al (2024) Synthesis and physical properties of polyhydroxyalkanoate (PHA)-based block copolymers: a review. Int J Biol Macromol 263:130204. 10.1016/j.ijbiomac.2024.130204 38365154 10.1016/j.ijbiomac.2024.130204

[14] McAdam B, Brennan Fournet M, McDonald P, Mojicevic M (2020) Production of polyhydroxybutyrate (PHB) and factors impacting its chemical and mechanical characteristics. Polymers 12:2908. 10.3390/polym12122908 33291620 10.3390/polym12122908 PMC7761907

[15] Zhila N, Shishatskaya E (2018) Properties of PHA bi-, ter-, and quarter-polymers containing 4-hydroxybutyrate monomer units. Int J Biol Macromol 111:1019–1026. 10.1016/j.ijbiomac.2018.01.130 29360547 10.1016/j.ijbiomac.2018.01.130

[16] Jung H-R, Jeon J-M, Yi D-H et al (2019) Poly(3-hydroxybutyrate-co-3-hydroxyvalerate-co-3-hydroxyhexanoate) terpolymer production from volatile fatty acids using engineered *Ralstonia eutropha*. Int J Biol Macromol 138:370–378. 10.1016/j.ijbiomac.2019.07.091 31310788 10.1016/j.ijbiomac.2019.07.091

[17] Kurz WA, Shaw CH, Boisvenue C et al (2013) Carbon in Canada’s boreal forest—a synthesis. Environ Rev 21:260–292. 10.1139/er-2013-0041

[18] Mao C, Del Rio LF, Al Dajani WW, et al (2015) FPInnovations’ TMP-bio for lignocellulosic biomass: current state and scaling up. NWBC 2015 6th Nord, pp 84–91

[19] Yuan Z, Browne TC, Zhang X (2017) Biomass Fractionation Process for Bioproducts. US Patent No. 9,580,454. Washington, DC: 26

[20] Dietrich K, Oliveira-Filho ER, Dumont M-J et al (2020) Increasing PHB production with an industrially scalable hardwood hydrolysate as a carbon source. Ind Crop Prod 154:112703. 10.1016/j.indcrop.2020.112703

[21] Blunt W, Shah P, Vasquez V et al (2023) Biosynthesis and properties of polyhydroxyalkanoates synthesized from mixed C5 and C6 sugars obtained from hardwood hydrolysis. New Biotechnol 77:40–49. 10.1016/j.nbt.2023.06.005 10.1016/j.nbt.2023.06.005 37390901

[22] Braunegg G, Sonnleitner B, Lafferty RM (1978) A rapid gas chromatographic method for the determination of poly-β-hydroxybutyric acid in microbial biomass. Eur J Appl Microbiol Biotechnol 6:29–37. 10.1007/BF00500854

[23] Coral J, Karp SG, De Souza P, Vandenberghe L et al (2008) Batch fermentation model of propionic acid production by *Propionibacterium acidipropionici* in different carbon sources. Appl Biochem Biotechnol 151:333–341. 10.1007/s12010-008-8196-1 18386184 10.1007/s12010-008-8196-1

[24] Wang Z, Sun J, Zhang A, Yang S (2013) Propionic acid fermentation. In: Yang S, El-Enshasy HA, Thongchul N (eds) Bioprocessing technologies in biorefinery for sustainable production of fuels, chemicals, and polymers, 1st edn. Wiley, New York, pp 331–350

[25] Gonzalez-Garcia R, McCubbin T, Navone L et al (2017) Microbial propionic acid production. Fermentation 3(2):21. 10.3390/fermentation3020021

[26] Falentin H, Deutsch S-M, Jan G et al (2010) The complete genome of *Propionibacterium freudenreichii* CIRM-BIA1T, a hardy actinobacterium with food and probiotic applications. PLoS ONE 5:e11748. 10.1371/journal.pone.0011748 20668525 10.1371/journal.pone.0011748 PMC2909200

[27] Zhou K, Yu J, Ma Y et al (2022) Corn steep liquor: green biological resources for bioindustry. Appl Biochem Biotechnol 194:3280–3295. 10.1007/s12010-022-03904-w 35349086 10.1007/s12010-022-03904-w

[28] Vidra A, Németh Á (2017) Bio-produced propionic acid: a review. Period Polytech Chem Eng 62:57–67. 10.3311/PPch.10805

[29] Pilevar Z, Mousavi Khaneghah A, Hosseini H, Ranaei V (2020) Propionic acid: method of production, current state and perspectives. Food Technol Biotechnol 58:115–127. 10.17113/ftb.58.02.20.6356 32831564 10.17113/ftb.58.02.20.6356 PMC7416123

[30] Deborde C, Boyaval P (2000) Interactions between pyruvate and lactate metabolism in *Propionibacterium freudenreichii* subsp. *shermanii*. In vivo13 C nuclear magnetic resonance studies. Appl Environ Microbiol 66:2012–2020. 10.1128/AEM.66.5.2012-2020.2000 10788375 10.1128/aem.66.5.2012-2020.2000 PMC101448

[31] Zhang A, Yang S-T (2009) Propionic acid production from glycerol by metabolically engineered *Propionibacterium acidipropionici*. Process Biochem 44:1346–1351. 10.1016/j.procbio.2009.07.013

[32] Wang P, Jiao Y, Liu S (2014) Novel fermentation process strengthening strategy for production of propionic acid and vitamin B12 by *Propionibacterium freudenreichii*. J Ind Microbiol Biotechnol 41:1811–1815. 10.1007/s10295-014-1513-5 25261985 10.1007/s10295-014-1513-5

[33] Wang Z, Jin Y, Yang S (2015) High cell density propionic acid fermentation with an acid tolerant strain of *Propionibacterium acidipropionici*. Biotech Bioeng 112:502–511. 10.1002/bit.25466 10.1002/bit.25466 25257628

[34] Bertrand J-L, Ramsay BA, Ramsay JA, Chavarie C (1990) Biosynthesis of poly-β-hydroxyalkanoates from pentoses by *Pseudomonas pseudoflava*. Appl Environ Microbiol 56:3133–3138. 10.1128/aem.56.10.3133-3138.1990 16348320 10.1128/aem.56.10.3133-3138.1990 PMC184911

[35] Silva L (2000) Propionic acid metabolism and poly-3-hydroxybutyrate-co-3-hydroxyvalerate (P3HB-co-3HV) production by *Burkholderia* sp. J Biotechnol 76:165–174. 10.1016/S0168-1656(99)00184-4 10656331 10.1016/s0168-1656(99)00184-4

[36] Chang KC, Nagarajan N, Gan Y-H (2024) Short-chain fatty acids of various lengths differentially inhibit *Klebsiella pneumoniae* and *Enterobacteriaceae* species. mSphere 9:e00781–23. 10.1128/msphere.00781-23 38305176 10.1128/msphere.00781-23 PMC10900885

[37] Royce LA, Liu P, Stebbins MJ et al (2013) The damaging effects of short chain fatty acids on *Escherichia coli* membranes. Appl Microbiol Biotechnol 97:8317–8327. 10.1007/s00253-013-5113-5 23912117 10.1007/s00253-013-5113-5 PMC3757260

[38] Rocco CJ, Escalante-Semerena JC (2010) In *Salmonella enterica*, 2-methylcitrate blocks gluconeogenesis. J Bacteriol 192:771–778. 10.1128/JB.01301-09 19948794 10.1128/JB.01301-09 PMC2812455

[39] Pereira EM, Silva-Queiroz SR, Cabrera Gomez JG, Silva LF (2009) Disruption of the 2-methylcitric acid cycle and evaluation of poly-3-hydroxybutyrate-*co*-3-hydroxyvalerate biosynthesis suggest alternate catabolic pathways of propionate in *Burkholderia sacchari*. Can J Microbiol 55:688–697. 10.1139/W09-018 19767840 10.1139/w09-018

[40] Brämer CO, Silva LF, Gomez JGC et al (2002) Identification of the 2-methylcitrate pathway involved in the catabolism of propionate in the polyhydroxyalkanoate-producing strain *Burkholderia sacchari* IPT101^T^ and analysis of a mutant accumulating a copolyester with higher 3-hydroxyvalerate content. Appl Environ Microbiol 68:271–279. 10.1128/AEM.68.1.271-279.2002 11772636 10.1128/AEM.68.1.271-279.2002 PMC126583

[41] Gomez JGC, Rodrigues MFA, Alli RCP et al (1996) Evaluation of soil gram-negative bacteria yielding polyhydroxyalkanoic acids from carbohydrates and propionic acid. Appl Microbiol Biotechnol 45:785–791. 10.1007/s002530050763

[42] Gomez JGC, Fontolan V, Alli RCP et al (1997) Production of P3HB-co-3HV by soil isolated bacteria able to use sucrose. Rev Microbiol 28:43–48

[43] Mendonça TT, Gomez JGC, Buffoni E et al (2014) Exploring the potential of *Burkholderia sacchari* to produce polyhydroxyalkanoates. J Appl Microbiol 116:815–829. 10.1111/jam.12406 24279348 10.1111/jam.12406

[44] Ashby RD, Solaiman DKY, Nuñez A et al (2018) *Burkholderia sacchari* DSM 17165: a source of compositionally-tunable block-copolymeric short-chain poly(hydroxyalkanoates) from xylose and levulinic acid. Bioresour Technol 253:333–342. 10.1016/j.biortech.2017.12.045 29413997 10.1016/j.biortech.2017.12.045

[45] Cesário MT, Raposo RS, de Almeida MCMD et al (2014) Production of poly(3-hydroxybutyrate-co-4-hydroxybutyrate) by *Burkholderia sacchari* using wheat straw hydrolysates and gamma-butyrolactone. Int J Biol Macromol 71:59–67. 10.1016/j.ijbiomac.2014.04.054 24811901 10.1016/j.ijbiomac.2014.04.054

[46] Choi MH, Song JJ, Yoon SC (1995) Biosynthesis of copolyesters by *Hydrogenophaga pseudoflava* from various lactones. Can J Microbiol 41:60–67. 10.1139/m95-169

[47] Zhao Y, Rao Z, Xue Y et al (2015) Biosynthesis, property comparison, and hemocompatibility of bacterial and haloarchaeal poly(3-hydroxybutyrate-co-3-hydroxyvalerate). Sci Bull 60:1901–1910. 10.1007/s11434-015-0923-8

[48] Shang L, Fei Q, Zhang YH et al (2012) Thermal properties and biodegradability studies of poly(3-hydroxybutyrate-co-3-hydroxyvalerate). J Polym Environ 20:23–28. 10.1007/s10924-011-0362-9

[49] Dalton B, Bhagabati P, De Micco J et al (2022) A review on biological synthesis of the biodegradable polymers polyhydroxyalkanoates and the development of multiple applications. Catalysts 12:319. 10.3390/catal12030319

[50] El-Hadi A, Schnabel R, Straube E et al (2002) Correlation between degree of crystallinity, morphology, glass temperature, mechanical properties and biodegradation of poly (3-hydroxyalkanoate) PHAs and their blends. Polym Test 21:665–674. 10.1016/S0142-9418(01)00142-8

[51] Surendran A, Lakshmanan M, Chee JY et al (2020) Can polyhydroxyalkanoates be produced efficiently from waste plant and animal oils? Front Bioeng Biotechnol 8:169. 10.3389/fbioe.2020.00169 32258007 10.3389/fbioe.2020.00169 PMC7090169

[52] Cox MK (1995) Recycling BIOPOL–composting and material recycling. J Macromol Sci A 32:607–612. 10.1080/10601329508010274

[53] Agus J, Kahar P, Abe H et al (2006) Molecular weight characterization of poly[(R)-3-hydroxybutyrate] synthesized by genetically engineered strains of *Escherichia coli*. Polym Degrad Stab 91:1138–1146. 10.1016/j.polymdegradstab.2005.07.006

[54] Bocanegra JK, Da Cruz Pradella JG, Da Silva LF et al (2013) Influence of pH on the molecular weight of poly-3-hydroxybutyric acid (P3HB) produced by recombinant *Escherichia coli*. Appl Biochem Biotechnol 170:1336–1347. 10.1007/s12010-013-0257-4 23666612 10.1007/s12010-013-0257-4

[55] Blunt W, Sparling R, Gapes DJ et al (2018) The role of dissolved oxygen content as a modulator of microbial polyhydroxyalkanoate synthesis. World J Microbiol Biotechnol. 10.1007/s11274-018-2488-6 29971506 10.1007/s11274-018-2488-6

[56] Tsuge T (2016) Fundamental factors determining the molecular weight of polyhydroxyalkanoate during biosynthesis. Polym J 48:1051–1057. 10.1038/pj.2016.78

[57] Arikawa H, Sato S, Fujiki T, Matsumoto K (2016) A study on the relation between poly(3-hydroxybutyrate) depolymerases or oligomer hydrolases and molecular weight of polyhydroxyalkanoates accumulating in *Cupriavidus necator* H16. J Biotechnol 227:94–102. 10.1016/j.jbiotec.2016.04.004 27059479 10.1016/j.jbiotec.2016.04.004

[58] Vo MT, Ko K, Ramsay B (2015) Carbon-limited fed-batch production of medium-chain-length polyhydroxyalkanoates by a *phaZ* -knockout strain of *Pseudomonas putida* KT2440. J Ind Microbiol Biotechnol 42:637–646. 10.1007/s10295-014-1574-5 25563970 10.1007/s10295-014-1574-5

