# Supplementary material for: Production and characterization of poly(3-hydroxybutyrate-co-3-hydroxyvalerate) copolymers from a pre- fermented hardwood hydrolysate
Source: Bioprocess Biosyst Eng. 2025 Jul 18;48(10):1679–92. doi: 10.1007/s00449-025-03203-8 (PMC12460433; doi:10.1007/s00449-025-03203-8)
Supplement: Supplementary file 1 — Supplementary file1 (DOCX 247 KB) [file 449_2025_3203_MOESM1_ESM.docx]

**Production and characterization of poly(3-hydoxybutyrate-*co*-3-hydroxyvalerate) copolymers from a pre-fermented hardwood hydrolysate**

Warren Blunt^1†^, Purnank Shah^2^, Vinicio Vasquez^1^, Meng Wei Ye^1^, Christopher Doyle^1^, Yali Liu^1^, Sajjad Saeidlou^3^, and Fanny Monteil-Rivera^1*^

^1^Aquatic and Crop Resource Development Research Centre, National Research Council Canada, 6100 Royalmount Avenue, Montreal, Québec, Canada, H4P 2R2; ^2^FPInnovations, 570 Boulevard Saint-Jean, Pointe-Claire, Québec, Canada H9R 3J9; ^3^Automotive and Surface Transportation Research Centre, National Research Council Canada, 75 de Mortagne Boulevard, Boucherville, Québec, Canada, J4B 6Y4

^*^Author to whom correspondence should be addressed:

[Fanny.Monteil-Rivera@cnrc-nrc.gc.ca](mailto:Fanny.Monteil-Rivera@cnrc-nrc.gc.ca)

^†^Author present address: Department of Biosystems Engineering, University of Manitoba, Winnipeg, Manitoba, Canada, R3T 5V6, Email: Warren.Blunt@umanitoba.ca

**Author emails:** [Warren.Blunt@cnrc-nrc.gc.ca](mailto:Warren.Blunt@cnrc-nrc.gc.ca); [purnank.shah@fpinnovations.ca](mailto:purnank.shah@fpinnovations.ca); [Vinicio.Vasquez@cnrc-nrc.gc.ca](mailto:Vinicio.Vasquez@cnrc-nrc.gc.ca); [MengWei.Ye@cnrc-nrc.gc.ca](mailto:MengWei.Ye@cnrc-nrc.gc.ca); [Christopher.Doyle@cnrc-nrc.gc.ca](mailto:Christopher.Doyle@cnrc-nrc.gc.ca); [Yali.Liu@cnrc-nrc.gc.ca](mailto:Yali.Liu@cnrc-nrc.gc.ca); [Sajjad.Saeidlou@cnrc-nrc.gc.ca](mailto:Sajjad.Saeidlou@cnrc-nrc.gc.ca); [Fanny.Monteil-Rivera@cnrc-nrc.gc.ca](mailto:Fanny.Monteil-Rivera@cnrc-nrc.gc.ca)

**Supplementary Information**

**Fig S1** Mixed sugar uptake and VFA production from *P. acidipropionici* grown in MSM supplemented with 20 g L^-1^ CSS and TMP-Bio sugars supplemented at an intial total sugar concentration of ca. 70 g L^-1^ in a 1 L bioreactor. Symbols: ▲, acetate; ◆, propionate; ×, lactate; ○, glucose; ☐, xylose

**a**

**b**

**Fig S2** *P. sacchari* grown in MSM medium with 10 g L^-1^ glucose and supplemented with different concentrations of lactate. **a)** OD_600_ profile over a 72-h cultivation and **b)** end-point analysis of total dry biomass and PHA synthesis.

**a**

**b**

**Fig S3** *H. pseudoflava* grown in MSM medium with 10 g L^-1^ glucose supplemented with different concentrations of lactate. **a)** OD_600_ profile over a 72-h cultivation and **b)** end-point analysis of total dry biomass and PHA synthesis.

**a**

**b**

**Fig S4** *P. sacchari* grown in MSM medium with 10 g L^-1^ glucose supplemented with different concentrations of acetate. **a)** OD_600_ profile over a 72-h cultivation and **b)** end-point analysis of total dry biomass and PHA synthesis

**b**

**a**

**Fig S5** *H. pseudoflava* grown in MSM medium with 10 g L^-1^ glucose supplemented with different concentrations of acetate. **a)** OD_600_ profile over a 72-h cultivation and **b)** end-point analysis of total dry biomass and PHA synthesis

**a**

**b**

**Fig S6** Total biomass synthesis rate (measured as increase in OD_600_) as a function of concentration of either lactate, acetate, or propionate at different concentrations for **a)** *P. sacchari* and **b)** *H. pseudoflava*

**a**

**b**

**Fig. S7** PHA synthesis data from MIC test using *P. sacchari*. **a)** Total biomass and PHA titer; and **b)** intracellular PHA content and 3-HV content of the polymer

**b**

**Fig S8** PHA synthesis data from MIC test using *H. pseudoflava*. **a)** Total biomass and PHA titer; and **b)** intracellular PHA content and 3-HV content of the polymer


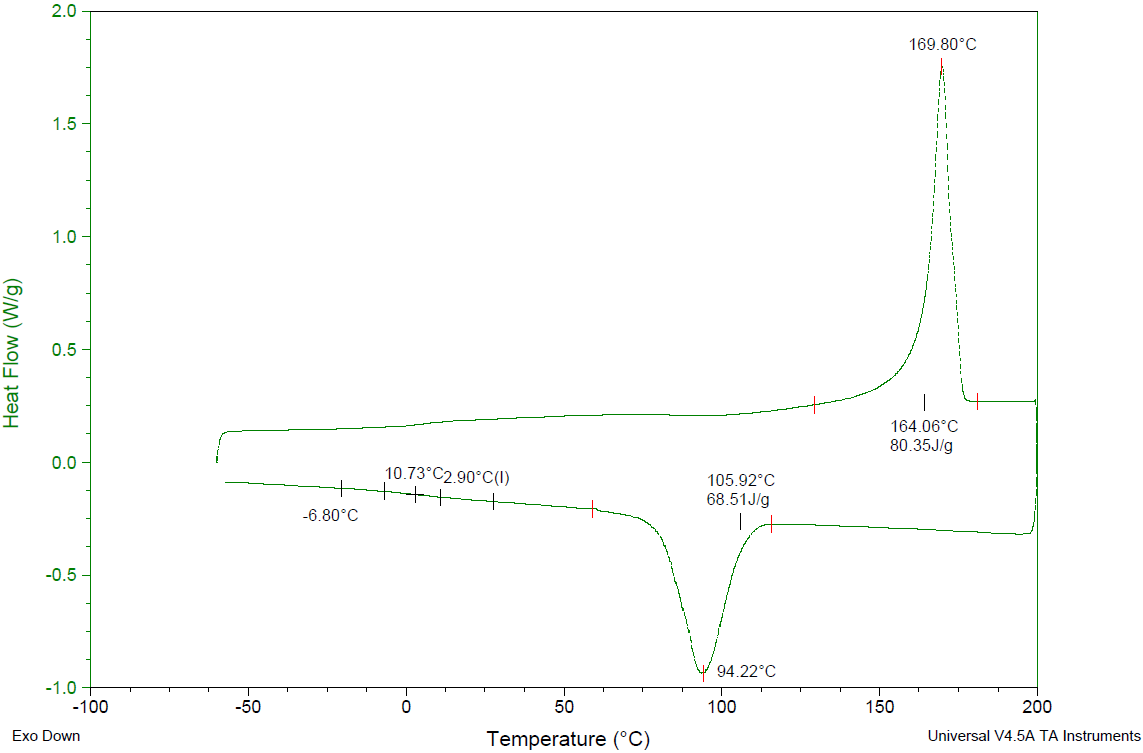


**Fig S9** Thermal profile obtained by differential scanning calorimetry (DSC) of a *P. sacchari* PHBV copolymer synthesized from pre-fermented TMP-Bio sugars and containing 1.2 mol% 3-HV.


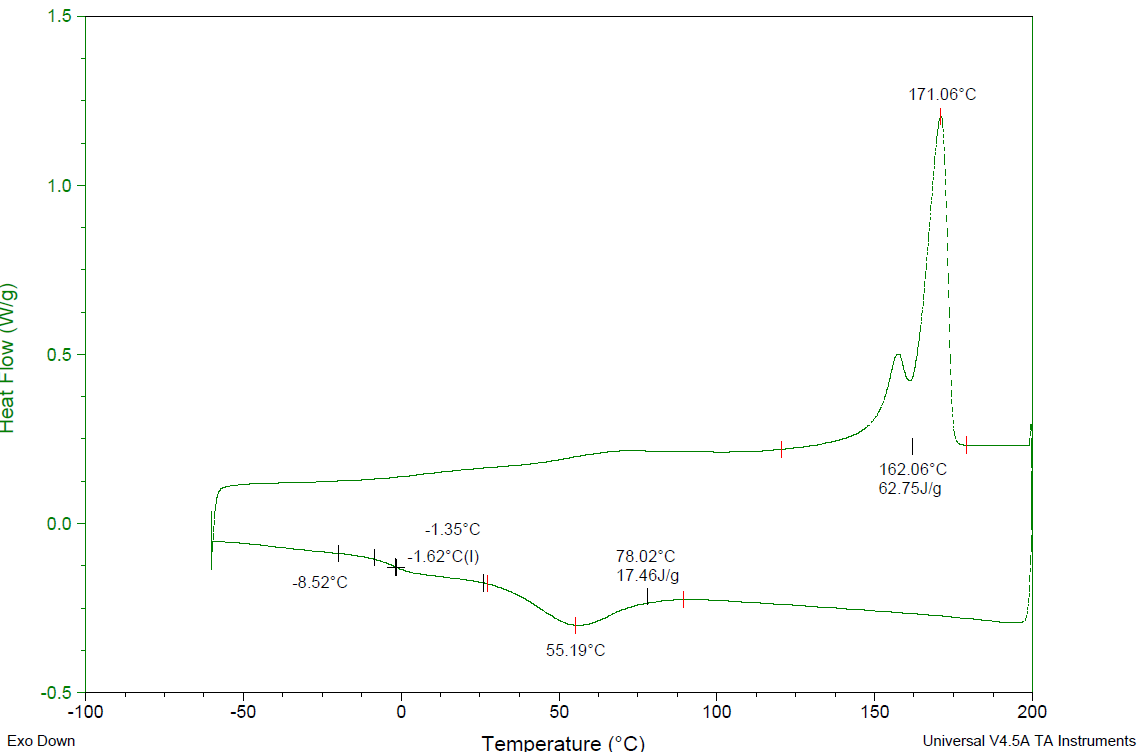


**Fig S10** Thermal profile obtained by differential scanning calorimetry (DSC) of a *P. sacchari* PHBV copolymer synthesized from pre-fermented TMP-Bio sugars and containing 5.1 mol% 3-HV.


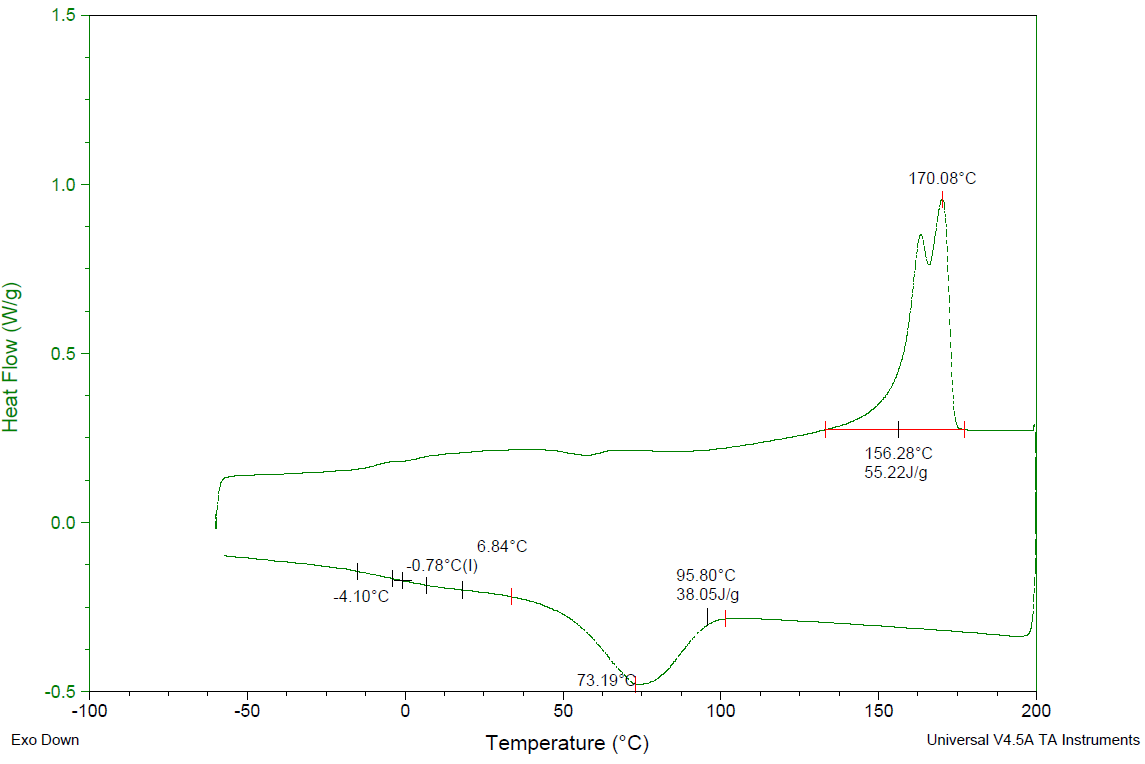


**Fig S11** Thermal profile obtained by differential scanning calorimetry (DSC) of a *H. pseudoflava* PHBV copolymer synthesized from pre-fermented TMP-Bio sugars and containing 13.7 mol% 3-HV.
